# Hsa-miR-214-3p inhibits breast cancer cell growth and improves the tumor immune microenvironment by downregulating B7H3

**DOI:** 10.32604/or.2024.057472

**Published:** 2024-12-20

**Authors:** YAN LU, KANG WANG, YUANHONG PENG, MENG CHEN, LIN ZHONG, LUJI HUANG, FU CHENG, XINDAN SHENG, XIN YANG, MANZHAO OUYANG, GEORGE A. CALIN, ZHIWEI HE

**Affiliations:** 1 China-America Cancer Research Institute, Guangdong Medical University, Dongguan, 523808, China; 2 GCP Center, Shunde Hospital, Southern Medical University (The First People’s Hospital of Shunde Foshan), Shunde, 528300, China; 3 Department of Gastrointestinal Surgery, Shunde Hospital, Southern Medical University (The First People’s Hospital of Shunde Foshan), Shunde, 528300, China; 4 The Second School of Clinical Medicine, Southern Medical University, Guangzhou, 510080, China; 5 Department of Translational Molecular Pathology, The University of Texas MD Anderson Cancer Center, Houston, TX 77030, USA; 6 Department of Oncology, Shunde Hospital, Southern Medical University (The First People’s Hospital of Shunde Foshan), Shunde, 528300, China

**Keywords:** Breast cancer, B7H3, Hsa-miR-214-3p, Immunotherapy

## Abstract

**Background:**

Immune checkpoint inhibitors play an important role in the treatment of solid tumors, but the currently used immune checkpoint inhibitors targeting programmed cell death-1 (PD-1), programmed cell death ligand-1 (PD-L1), and cytotoxic T-lymphocyte antigen-4 (CTLA-4) show limited clinical efficacy in many breast cancers. B7H3 has been widely reported as an immunosuppressive molecule, but its immunological function in breast cancer patients remains unclear.

**Methods:**

We analyzed the expression of B7H3 in breast cancer samples using data from the Cancer Genome Atlas Program (TCGA) and the Gene Expression Omnibus (GEO) databases. MicroRNAs were selected using the TarBase, miRTarBase, and miRBase databases. The regulatory role of the microRNA hsa-miR-214-3p on B7H3 was investigated through dual-luciferase reporter assays, which identified the specific action sites of interaction. The expression levels of B7H3 and hsa-miR-214-3p in human breast cancer tissues and adjacent normal tissues were quantified using Western blotting and quantitative PCR (qPCR). *In vitro* experiments were performed to observe the effects of modulating the expression of B7H3 or hsa-miR-214-3p on breast cancer cell proliferation and apoptosis. Additionally, the regulatory impact of hsa-miR-214-3p on B7H3 was examined. Enzyme-linked immunosorbent assays (ELISA) and flow cytometry were employed to assess the effects of co-cultured breast cancer cells and normal human peripheral blood mononuclear cells (PBMCs) on immune cells and associated cytokines.

**Results:**

In breast cancer tissues, the expression level of B7H3 is inversely correlated with that of hsa-miR-214-3p, as well as with the regulatory effects on breast cancercell behavior. Hsa-miR-214-3p was found to inhibit breast cancer cell growth by downregulating B7H3. Importantly, our research identified, for the first time, two binding sites for hsa-miR-214-3p on the 3’ UTR of B7H3, both of which exert similar effects independently. Co-culture experiments revealed that hsa-miR-214-3p obstructs the suppressive function of B7H3 on CD8^+^ T cells and natural killer cells.

**Conclusions:**

This study confirms the existence of two hsa-miR-214-3p binding sites on the 3’ UTR of B7H3, reinforcing the role of hsa-miR-214-3p as a regulatory factor for B7H3. In breast cancer, hsa-miR-214-3p reduces tumor cell proliferation and enhances the tumor immune microenvironment by downregulating B7H3. These findings suggest new potential targets for the clinical treatment of breast cancer.

## Introduction

Breast cancer is a primary cause of cancer incidence, disability, and mortality among women globally [1], An analysis of worldwide data by the American Cancer Society indicates that breast cancer has now surpassed lung cancer as the most prevalent cancer in women [2]. Evidence from the American Cancer Society suggests that both past and ongoing research have significantly enhanced survival rates for breast cancer patients, primarily due to advances in screening, diagnostic, and treatment methodologies. Despite these advancements, the prognosis for advanced breast cancer remains dire, with many patients developing resistance to treatment. Immunotherapy has emerged as a novel therapeutic approach for advanced breast cancer; however, it proves ineffective for a significant number of patients. Consequently, identifying new targets is imperative to enhance the efficacy of immunotherapy in breast cancer treatment.

B7H3, also known as CD276, is the most recently identified member of the B7 family of ligands. It is a type-I transmembrane glycoprotein consisting of extracellular, transmembrane, and short intracellular domains [3]. B7H3 exhibits low expression in most normal tissues but is overexpressed in a variety of solid tumors, including melanoma, glioma, lung cancer, pancreatic cancer, ovarian cancer, breast cancer, gastric cancer, and again particularly in breast cancer [4]. Its expression varies among different tumors, possibly due to the unidentified receptor molecule associated with B7H3. Identifying this receptor molecule could elucidate the variations in B7H3 expression across different tumors [5]. Initially characterized as a co-stimulatory molecule for T-cell activation [6], subsequent research has shown that B7H3 predominantly exerts a potent immunosuppressive effect, inhibiting T-cell proliferation. It has been demonstrated that B7H3 protein expression can be induced on the surface of immune cells such as dendritic cells, monocytes, and B cells [6]. B7H3 plays a crucial role in modulating the immune response of T cells, negatively regulating the activity of key transcription factors including the nuclear factor of activated T-cells (NFAT), activator protein-1 (AP-1), and nuclear factor κB (NF-κB). Additionally, B7H3 reduces interleukin-2 (IL-2) production, impairs the proliferation and activation of CD4^+^ and CD8^+^ T cells, and suppresses Th1 cells and interferon-γ (IFN-γ) production, which may exacerbate autoimmune encephalomyelitis [5,7]. Research indicates that in breast cancer, B7H3 expression impedes the proliferation of CD4^+^ and CD8^+^ T cells by diminishing the phosphorylation of the mammalian target of rapamycin (mTOR), thereby curtailing the release of IFN-γ. Thus, B7H3 represents a promising target for breast cancer immunotherapy. A deeper understanding of these intricate immune-regulatory mechanisms could aid in controlling breast cancer progression and potentially refining immunotherapeutic strategies.

MicroRNAs (miRNAs) are noncoding RNAs, approximately 19–24 nucleotides in length, that regulate gene expression either by degrading mRNA or inhibiting transcription. They achieve this by binding to the 3’ untranslated regions of their target mRNAs. Increasingly, research has shown that miRNAs play a critical role in tumor development by modulating the tumor microenvironment. For instance, Jia et al. demonstrated that overexpression of miR-142-5p inhibits the PD-1/PD-L1 pathway, thereby enhancing antitumor immune functions [8]. Additionally, studies have shown that hsa-miR-214-3p suppresses retinoblastoma cell proliferation by targeting the PI3K/AKT/mTOR pathway [9]. In non-small cell lung cancer, NCAPG expression is associated with increased cell proliferation, migration, and self-renewal capacities; however, overexpression of hsa-miR-214-3p can counter these effects [10]. Contrasts in serum miR-214-3p levels between breast cancer patients and healthy controls have been significant [11]. Hsa-miR-214-3p also reduces proliferation and promotes apoptosis in breast cancer cells by regulating survivin expression [12]. When miR-214-3p inhibitors are transfected into MCF-7 breast cancer cells, their proliferative capacity increases, whereas transfection with miR-214-3p mimics reduces it. Thus, hsa-miR-214-3p is pivotal in breast cancer development, warranting further investigation into its regulatory mechanisms. Given B7H3’s role in the immune evasion of breast cancer, it is hypothesized that hsa-miR-214-3p could suppress breast cancer cell proliferation and enhance immune cell activity by modulating B7H3 expression, thereby influencing BRCA development.

Considering B7H3’s significant influence on tumor immunity, the mechanisms regulating its expression in breast cancer, particularly upstream, remain unclear. Our research focuses on B7H3 as a starting point to identify upstream regulatory targets. We have linked miRNA and B7H3 to explain variations in B7H3 protein expression between normal and tumor tissues, exploring post-transcriptional modulation pathways and how B7H3 regulates tumor immunity. As a target of hsa-miR-214-3p, this miRNA inhibits breast cancer cell proliferation through the regulation of B7H3 expression, restores the activity and proliferation of CD8^+^ T cells and natural killer (NK) cells, and promotes the production of immune factors such as IL-2, IL-4, and IFN-γ, thereby mitigating breast cancer development.

## Materials and Methods

### Breast cancer tissues, cell lines, and cell culture

Breast cancer and adjacent noncancerous tissue samples were obtained from 20 patients with breast cancer admitted to Shunde Hospital, Southern Medical University since 01 January 2021. Pathological stage, treatment, and drug resistance were recorded for all patients. All clinical samples were stored using cryopreservation in liquid nitrogen tanks after rapid freezing for 40 min *in vitro*, and all tissue samples used for total RNA extraction were supplemented with RNA protectants. Use of all samples was approved by the Ethics Committee at the Medical Research Ethics Committee of Shunde Hospital, Southern Medical University (KYLS20221117, 2022-11-02), and informed consent was obtained from the patients before sample collection in accordance with Declaration of Helsinki.

All breast cancer cell lines used in this study were obtained from the cell bank of the Chinese Academy of Sciences. The breast cancer cell lines MCF-7, BT-474, SUM-149, and MDA-231 were cultured in high-sugar Dulbecco’s modified Eagle’s medium (C11995500BT, Gibco, Grand Island, NY, USA) containing 10% fetal bovine serum (A5670701, Gibco) with 100 μg/mL streptomycin and 100 IU/mL penicillin (15140122, Gibco). All cell lines were cultured in a 37°C, 5% CO_2_, 95% humidity incubator.

### Bioinformatic analysis

High-throughput RNA sequencing data (Workflow Type: HTseq-FPKM, Fragments Per Kilobase per Million) and corresponding clinical and pathological data from the breast cancer project, TCGA-BRCA, were downloaded from The Cancer Genome Atlas (TCGA) database (https://portal.gdc.cancer.gov/) (accessed on 18 November 2024). The RNA sequencing data were converted to TPM (transcription per million reads). The research method was conducted entirely according to TCGA guidelines. Also, the Gene Expression Omnibus (GEO) database (http://www.ncbi.nlm.nih.gov/geo/) (accessed on 18 November 2024) was searched for breast cancer mRNA expression data and clinical data as an external validation of survival analyses. The inclusion criteria data set should include breast cancer sample groups and control sample groups (healthy tissue, adjacent noncancerous tissue), and non-cancerous tissue and cancer tissue should contain at least 10 samples. The GSE16201 gene expression profile was selected from the GEO database based on the above inclusion criteria.

The Wilcoxon rank sum test (continuous variables) or Pearson chi-square test was used to compare the clinical and pathological characteristics of groups of breast cancer patients from TCGA database with high and low B7H3 expression. The correlation of B7H3 expression and clinical and pathological features was evaluated using logistic analysis. Using receiver operating characteristic analysis, the expression of B7H3 in breast cancer and adjacent tissue samples was compared, and the predictive value of B7H3 in the diagnosis of breast cancer was tested. The R package DESeq2 (version 1.26.0) was used to identify differentially expressed genes in expression profiles (HTSeq-TPM) between the high and low B7H3 mRNA expression groups, and a heat map was created to visualize the result using the R package ggplot2 (version 3.3.3, Ross Ihaka and Robert Gentleman from the University of Auckland in New Zealand). The molecular interactions of the B7H3 protein list with miRNAs were analyzed to construct a protein-protein interaction network through using the STRING database (https://cn.string-db.org/) (accessed on 18 November 2024) and visualized the results using the R package igraph (version 3.3.3). Gene ontology based functional annotation and Kyoto Encyclopedia of Genes and Genomes pathway enrichment analysis of B7H3 were performed. The clusterProfiler R package (version 3.14.3) was used for pathway enrichment analysis, whereas ggplot2 (version 3.3.3) was used for pathway visualization. Gene set enrichment analysis was also employed using clusterProfiler in order to elucidate the significant function and pathway differences between the high- and low-B7H3 groups. The single-sample gene set enrichment analysis algorithm in the R package GSVA (version 3.3.3) was to perform immune infiltration analysis of B7H3; in total, 24 invasive immune cells were identified and visualized using R package ggplot2 (version 3.3.3). Spearman’s rank correlation coefficient was used to analyze the correlation between B7H3 and the 24 invasive immune cell enrichment scores. The Wilcoxon rank sum test was used to analyze the B7H3 enrichment scores in the high- and low-B7H3 expression groups of samples. R software (version 3.3.3) was used for the normalization, analysis, and visualization of data.

### Dual luciferase reporter gene assay

A dual luciferase reporter gene assay was used to confirm that B7H3 is the direct target of hsa-miR-214-3p. The wild-type and three mutated sequences of B7H3 mRNA were cloned into plasmids (HanBio Therapeutics, Shanghai, China). The luciferase constructs described above were co-transfected with hsa-miR-214-3p mimics or normal control (NC) mimics into 293 T cells, and an empty vector was transfected into 293 T cells as the control group. After 48 h of transfection, the cells were harvested, and the light generated by intracellular luciferase was detected and analyzed using a dual luciferase reporter gene assay system (16185, Gibco).

Sequence of target gene for dual-luciferase reporter experiment: **h-B7H3-3UTR-wt:** CCATGAGGACCAGGGAGCTGCTACCCCTCCCTACAGCTCCTACCCTCTGGCTGCAATGGGGCTGCACTGTGAGCCCTGCCCCCAACAGATGCATCCTGCTCTGACAGGTGGGCTCCTTCTCCAAAGGATGCGATACACAGACCACTGTGCAGCCTTATTTCTCCAATGGACATGATTCCCAAGTCATCCTGCTGCCTTTTTTCTTATAGACACAATGAACAGACCACCCACAACCTTAGTTCTCTAAGTCATCCTGCCTGCTGCCTTATTTCACAGTACATACATTTCTTAGGGACACAGTACACTGACCACATCACCACCCTCTTCTTCCAGTGCTGCGTGGACCATCTGGCTGCCTTTTTTCTCCAAAAGATGCAATATTCAGACTGACTGACCCCCTGCCTTATTTCACCAAAG; **h-B7H3-3UTR-mu1:** CCATGAGGACCAGGGAGCTGCTACCCCTCCCTACAGCTCCTACCCTCTGGCTGCAATGGGGCTGCACTGTGAGCCCTGCCCCCAACAGATGCATCCTGCTCTGACAGGTGGGCTCCTTCTCCAAAGGATGCGATACACAGACCACTGTGCAGCCTTATTTCTCCAATGGACATGATTCCCAAGTCATGGTCGACCCTTTTTTCTTATAGACACAATGAACAGACCACCCACAACCTTAGTTCTCTAAGTCATCCTGCCTGCTGCCTTATTTCACAGTACATACATTTCTTAGGGACACAGTACACTGACCACATCACCACCCTCTTCTTCCAGTGCTGCGTGGACCATCTGGCTGCCTTTTTTCTCCAAAAGATGCAATATTCAGACTGACTGACCCCCTGCCTTATTTCACCAAAG; **h-B7H3-3UTR-mu2:** CCATGAGGACCAGGGAGCTGCTACCCCTCCCTACAGCTCCTACCCTCTGGCTGCAATGGGGCTGCACTGTGAGCCCTGCCCCCAACAGATGCATCCTGCTCTGACAGGTGGGCTCCTTCTCCAAAGGATGCGATACACAGACCACTGTGCAGCCTTATTTCTCCAATGGACATGATTCCCAAGTCATCCTGCTGCCTTTTTTCTTATAGACACAATGAACAGACCACCCACAACCTTAGTTCTCTAAGTCATCCTGGGTCGACCCTTATTTCACAGTACATACATTTCTTAGGGACACAGTACACTGACCACATCACCACCCTCTTCTTCCAGTGCTGCGTGGACCATCTGGCTGCCTTTTTTCTCCAAAAGATGCAATATTCAGACTGACTGACCCCCTGCCTTATTTCACCAAAG; **h-B7H3-3UTR-mu3:** CCATGAGGACCAGGGAGCTGCTACCCCTCCCTACAGCTCCTACCCTCTGGCTGCAATGGGGCTGCACTGTGAGCCCTGCCCCCAACAGATGCATCCTGCTCTGACAGGTGGGCTCCTTCTCCAAAGGATGCGATACACAGACCACTGTGCAGCCTTATTTCTCCAATGGACATGATTCCCAAGTCATGGTCGACCCTTTTTTCTTATAGACACAATGAACAGACCACCCACAACCTTAGTTCTCTAAGTCATCCTGGGTCGACCCTTATTTCACAGTACATACATTTCTTAGGGACACAGTACACTGACCACATCACCACCCTCTTCTTCCAGTGCTGCGTGGACCATCTGGCTGCCTTTTTTCTCCAAAAG ATGCAATATTCAGACTGACTGACCCCCTGCCTTATTT CACCAAAG; **hsa-miR-214-3p MIM AT0000271:** ACAGC AGGCACAGACAGGCAGU.

### Real-time PCR

Total RNA was extracted from breast cancer tissue samples and cells using a total RNA extraction kit (Vazyme, R401-01, Nanjing, China), and reverse transcription (RT)-quantitative polymerase chain reaction (qPCR) analysis of the RNA was performed using a Bio-Rad CFX96 real-time PCR instrument (Bio-Rad, Hercules, CA, USA), reverse transcription kit (R222-01, Vazyme, Nanjing, China), miRNA reverse transcription kit (MR101-02, Vazyme), and ChamQ Universal SYBR qPCR Master Mix kit (Q711-03, Vazyme). MiRNA was amplified using the stem-loop method. All experiments were conducted according to the standard experimental scheme that have been published previously [13] using β-actin as the gene reference, U6 as the miRNA reference, and primers synthesized by Generay Biotech (Shanghai, China). The primer sequences were 5’-CTACCTCATGAAGATCCTCACCGA-3’ (β-actin, forward), 5’-TTCTCCTTAATGTCACGCACGATT-3’ (β-actin, reverse), 5’-CTCGCTTCGGCAGCACA-3’ (U6, forward), 5’-AACGCTTCACGAATTTGCGT-3’ (U6, reverse), 5’-CGGGCACAGCAGGCACAGAC-3’ (hsa-miR-214-3p, forward), 5’-CAGCCACAAAAGGCACAAT-3’(hsa-miR-214-3p, reverse), CCTGTTGTCTCCAGCCACAAAAGAGCACAATATTTCAGGAGACAACAGGACTGCCT (stem sequence for RT), 5’-AGGGCAGCCTATGACATTCC-3’ (B7H3, forward), and 5’-ACCAGCAGTGCAATGAGACA-3’ (B7H3, reverse).

### Fluorescence *in situ* hybridization assay AND immunofluorescence

The specific FISH probe used is a 5’-FAM labled RNA oligonucleotide synthesised by Beijing Tsingke Biotech Co., Ltd., Beijing, China, targeting the full length of miRNA-214-3p. The probe sequence is the reverse complementary sequence of miRNA-214-3p: 5’-FAM-ACUGCCUGUCUGUGCCUGCUGU-3’, Its specificity is determined by alignment in the NCBI (https://blast.ncbi.nlm.nih.gov/Blast.cgi) (accessed on 18 November 2024).

Cells in mid-log phase are washed 2 times using D-PBS and fixed by 4% paraformaldehyde (BL539A, Biosharp, Hefei, China) for for 10 min on ice. After fixation and a subsequent 5 min washing in D-PBS, cells are incubated in ice-cold 100% methanol for 1 h at 4°C to permeablize them and remove pigments. For subsequent steps, follow the protocol of Fluorescence *in situ* Hybridization Kit for RNA (R0306S, Beyotime, Shanghai, China). Cells are treated with 0.5 M HCl and Acetylation Solution for neutralizing alkaline proteins and deacetylation. For pre-hybridization, incubate sample with yeast RNA-containing (1X) hybridization solution at 50°C for 20 min, remove the hybridization solution and wash with D-PBS 2 times. Add the probe-containing (1 μg/mL) hybridization fluid to immerse the sample, seal, and then place it on a shaker in the dark at 50°C for hybridization for 10 h. After washing by Washing BufferI/II/III (each for 10 min at 50°C) successively, nucleus was stained by DAPI (1 μg/mL) in the dark for 3–5 min.

Following fixation, the cells were washed in (DEPC)-treated PBS and incubated in ice-cold 100% methanol for 1 h at 4°C to permeablize and remove pigments that might interfere with the fluorescence signal of the secondary antibody. Cells were washed in DEPC-treated PBS containing 0.5% Tween 20 (D-PBST) and incubated for 45 min at room temperature with rotation in D-PBST containing 3% BSA (V900933-100G, Sigma-Aldrich, St. Louis, MO, USA) as a blocking agent. Samples were then incubated overnight at 4°C with rotation in D-PBST containing the primary polyclonal rabbit anti-CD276 antibody (14453-1-AP, proteintech, 1:500). Following primary antibody incubation, samples were washed twice in D-PBST with rotation for 5 min, and were incubated on ice for 1 h in the dark with the secondary antibody, Cy3–conjugated Goat Anti-Rabbit IgG(H + L) (SA00009-2, Proteintech, 1:100). Samples were washed once in D-PBST and once in D-PBS for 10 min each with rotation at RT.

### Western blot analysis

Total protein was extracted from breast cancer tissue samples and cells using a mixture of RIPA lysate (P0013B, Beyotime) and a protease inhibitor (P1005, Beyotime; 100:1). The above protein concentration was determined using BCA (P0012, Beyotime). After boiling and denaturing at 100°C, the protein was placed on ice, and a polyacrylamide gel electrophoresis gel at a concentration of 8% was prepared for electrophoresis. After electrophoresis, the membranes were transferred and sealed, and the membranes were incubated with primary anti-B7H3 (30052-1-AP, 1:1000, Proteintech, Wuhan, China) and anti-β-actin (Proteintech; 66009-1-Ig, 1:1000) antibodies. The membranes were incubated overnight at 4°C in a shaker. After washing the membranes, the membranes were incubated with the corresponding rabbit (Proteintech; SA00001-2-100UL, 1:3000) and mouse (Proteintech; SA00001-1-100UL, 1:3000) secondary antibodies. After incubation at room temperature for 2 h, exposure and development of the membranes were performed using a Bio-Rad chemiluminescence instrument (Bio-Rad, ChemiDoc MP Imaging System, Hercules, CA, USA). Grayscale analysis for WB strips were performed using ImageJ software (National Institutes of Health, ImageJ).

### Cell counting Kit-8 (CCK-8) proliferation assay

MCF-7 or BT-474 cells from different treatment groups were inoculated into a 96-well plate with 3000–5000 cells per well and placed in a culture incubator. Time-gradient cultivation of the cells was performed for 24–96 h, and 90 μL of culture medium and 10 μL of CCK-8 reagent (GlpBio, GK10001-1, Monrovia, CA, USA) were added to each well every 24 h. After the above treatment, the cells were cultured in a cell incubator in the dark was performed for 2.5 h, and a multifunctional enzyme-labeling instrument (VICTOR Nivo™, Perkin-Elmer, Waltham, MA, USA) was used to detect the absorbance of the cells at 450 nm. Prism software (version 8.2.0, GraphPad Software, San Diego, CA, USA) was used for statistical analysis and graphic rendering.

### Plate clone proliferation assay

Different treatment groups of MCF-7 or BT-474 cells at the logarithmic growth phase were inoculated at 1000 cells per dish into 6 cm × 6 cm dishes, which was followed by gentle rotation to disperse the cells evenly. The cells were incubated for 2–3 weeks. When visible clones appeared in a culture dish, the culture was terminated. The supernatant was discarded, and the cells were carefully soaked twice with PBS. The cells were fixed in 1 mL of methanol for 15 min, the fixative was removed from the dishes, an appropriate amount of Giemsa staining solution (LEAGENE, DM0012, Beijing, China) was added to the dishes for 30 min, the staining solution was slowly washed off with running water, and the cells were air-dried. Next, the plate was inverted and overlaid on a transparent film with a grid, and the clones were counted directly with the naked eye or under a microscope (Leica, DMi1 Inverted Microscope for Cell Culture) (low magnification) for clones of more than 10 cells. Finally, (the formula number of clones)/(number of inoculated cells) × 100% was used to calculate the clone formation rate.

### EdU cell proliferation assay

An EdU detection kit (Beyotime; C0071L) was used for assessing the proliferative activity of cells. A total of 1 × 10^6^ breast cancer cells was inoculated into a six-well plate and transfected with different plasmid or siRNA according to different treatment groups. After 36 h of culture, 2 μM EdU buffer was added to the plate, and the cells were incubated at 37°C for one tenth of the cell cycle. After labeling the cells with EdU, the culture medium was removed, and 1 mL of 4% paraformaldehyde was added to the plate to fix the cells at room temperature for 15 min. The fixing solution was then removed from the plate, and the cells were washed thoroughly. Next, the washing solution was removed from the plate, the cells were treated with 1 mL of 0.3% Triton X-100 permeable solution per well, and the cells were incubated at room temperature for 10 min. The transparent liquid was then removed from the plate, and the cells were washed thoroughly. Click reaction solution was added to the culture medium, and the nuclei were stained with Hoechst 33342 (62249, Gibco). Subsequently, fluorescence detection in cells was performed. Hoechst 33342 exhibits blue fluorescence with a maximum excitation wavelength of 346 nm and maximum emission wavelength of 460 nm.

### Cell-cycle assay

After culture to the logarithmic growth stage, BT-474 cells were inoculated at 2 × 10^5^ cells per well in a six-well plate. After 24 h, the cells were fully adherent to the wells of the plate and transfected with different plasmid or siRNA according to different treatment groups. They were then divided into wild-type, hsa-miR-214-3p-NC, hsa-miR-214-3p-mimics, B7H3-overexpressing with hsa-miR-214-3p-NC (OE-B7H3 + hsa-miR-214-3p-NC), and OE-B7H3 + hsa-miR-214-3p-mimics groups, and the culture was continued for 48 h. Cells were collected from the plate and stained using a cell cycle detection kit (KeyGen Biotech, KGA9101-100, Nanjing, China). Stained cells were collected using a FACSCanto instrument (BD Biosciences, San Jose, CA, USA), and the results of different groups were analyzed using Prism software (version 8.2.0).

### Flow cytometry assay

In a co-culture experiment with human peripheral blood mononuclear cells (PBMCs) and breast cancer cells, BT-474 cells were cultured in six-well, round-bottom culture plates at 2 × 10^5^ cells per well, and the cell adhesion to the wells of the plate was observed. After 24 h, the cells were divided into wild-type, hsa-miR-214-3p-NC, hsa-miR-214-3p-mimics, OE-B7H3 + hsa-miR-214-3p-NC, and OE-B7H3 + hsa-miR-214-3p-mimics, groups and transfected with corresponding plasmid or mimics. After 24 h of transfection, PBMCs were extracted from normal human peripheral blood samples obtained from 10 healthy volunteers admitted to Shunde Hospital, Southern Medical University, resuspended in RPMI 1640 medium (C11875500BT, Gibco) containing 10% FBS, and counted. Next, 1 × 10^6^ PBMCs were added to the precultured six-well, round-bottom cell culture plates (PBMCs: tumor cell ratio, 1 × 10^6^:2 × 10^5^ [5:1]), and activator Human CD28 Activating Mouse mAb (91920S, Cell Signaling Technology, Danvers, MA, USA, 5 μg/mL) was added to each well. After 48 h of co-culture, the PBMCs supernatant suspension was collected and subjected to flow cytometric detection of various immune molecules. PBMCs were resuspended in 100 μL of PBS, fluorescently labeled antibodies (such as CD3, CD4, CD8, CD56, CD11b, etc.), were added to each tube, negative and positive control tubes were set, and changes in the CD3^+^CD4^+^, CD3^+^CD8^+^, CD3^-^CD56^+^, and CD11b^+^ cell proportions were analyzed using flow cytometry. Flow cytometric data were collected using the FACSCanto instrument and analyzed using FlowJo software (version 10, Becton, Dickinson and Company (BD), Franklin Lakes, NJ, USA).

### Enzyme-linked immunosorbent assay (ELISA)

PBMCs in the co-culture system described above were centrifuged, and the supernatant was collected. Standard from the ELISA kit, blank, and sample wells were set separately and added to enzyme-linked plates with precoated anti-cytokine antibodies (IL-2, IL-4, and IFN-γ) (Elabscience, E-EL-H0099c, E-EL-H0101c, E-EL-H0108c, Wuhan, China). One hundred microliters of diluted standard solution were added to the standard well, 100 μL of standard solution from the ELISA kit and sample diluent was added to the blank well, and 100 μL of the sample to be tested (the supernatant) was added to the sample wells. Following standard experimental procedures, the optical density values for each well were measured at a wavelength of 450 nm using the multifunctional enzyme-labeling instrument (VICTOR Nivo™, Perkin-Elmer, Waltham, MA, USA). Changes in the cytokine concentration in each group of the supernatant were analyzed.

### Statistical analysis

Prism software (version 8.2.0, GraphPad Software, San Diego, CA, USA) was used to perform statistical analyses. Both Western blot and RT-qPCR experimental data were subjected to paired two-sample *t*-tests, as were the CCK-8 experimental data. The EdU cell proliferation assay and plate clone proliferation assay were used to count the cells in the field of view in each well; an independent two-sample *t*-test was used for the cell number data of each group. Furthermore, a homogeneity of variance test was performed on each set of data before statistical analysis. If the variance was uneven, a *t*-test was used; if not, an approximate *t*-test was used. *p* < 0.05 was considered statistically significant.

## Results

### Bioinformatic analysis demonstrated that B7H3 was highly expressed in breast cancer tissues and hsa-miR-214-3p may be the binding site of B7H3

**Figure 1:**
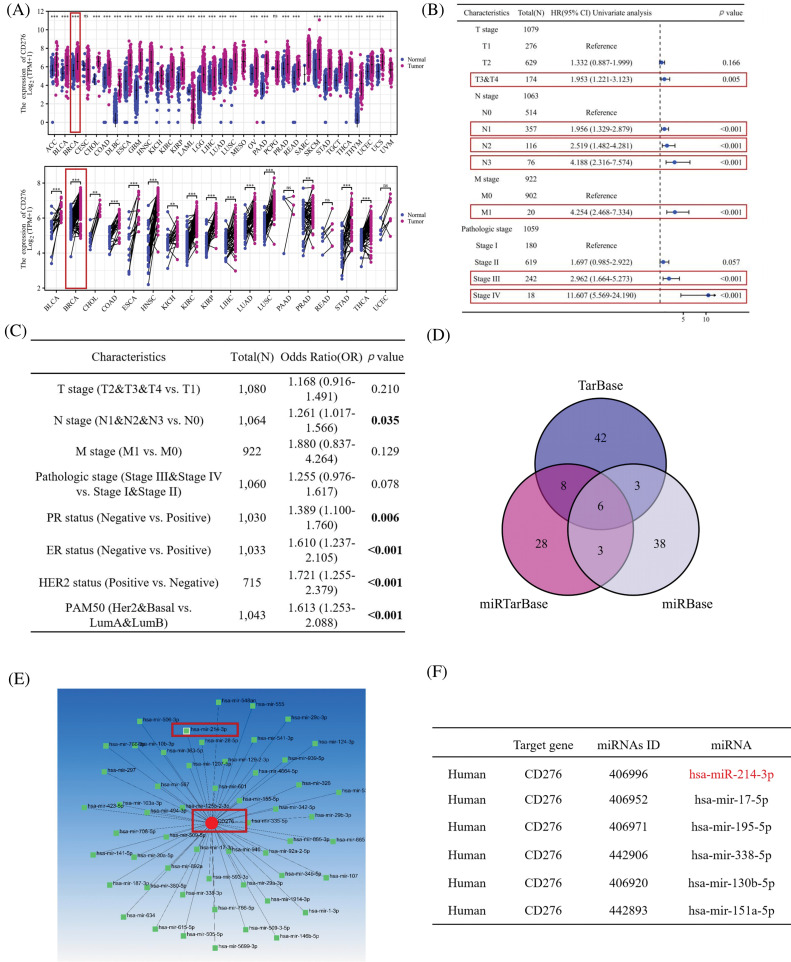
B7H3 is highly expressed in breast cancer samples, and its expression is associated with poor prognosis. Also, hsa-miR-214-3p is predicted to be a possible binding site for B7H3. (A) Expression of B7H3 in both unpaired and paired samples of different tumors from the Cancer Genome Atlas Program (TCGA) database. **p < 0.01; ***p < 0.001; ns, not significant. (B) Univariate Cox regression analysis of the relationship between B7H3 expression and clinical features in breast cancer patients in the TCGA database. (C) Logistic regression analysis of the relationship between B7H3 expression and different clinical features of breast cancer patients. (D) Venn diagram of the possible binding miRNAs for B7H3 identified in the TarBase, miRTarBase, and miRBase databases. (E) Diagram of the B7H3 (CD276)/miRNA interaction network. (F) The top six miRNAs with the highest matching degree to the binding site of the 3’-UTR region of B7H3.

Analysis of TCGA data demonstrated that B7H3 mRNA expression levels were high in different tumors. As shown in Fig. 1A, B7H3 was significantly overexpressed in 27 out of 33 tumors and was also highly expressed in breast cancer tissues. Univariate Cox regression analysis demonstrated that B7H3 had a high hazard ratio, similar to that of clinical TNM stage and pathological grade (Fig. 1B). At the same time, we carried out logistic regression analysis on B7H3 and compared different clinical characteristics of breast cancer patients, such as TNM stage, pathological grade, and progesterone receptor, estrogen receptor, HER2, and PAM50 status. Among breast cancer patients, we found that B7H3 expression has a high odds ratio in N stage. Except that, B7H3 expression was high in patients with common immunohistochemical indicators of breast cancer such as progesterone receptor (*p* = 0.006), estrogen receptor (*p* < 0.001), HER2 (*p* < 0.001), and PAM50 (*p* < 0.001). This demonstrated that B7H3 is clinically important in breast cancer cases (Fig. 1C).

Subsequently, we identified the possible binding sites for B7H3 through searching multiple databases (TarBase: https://dianalab.e-ce.uth.gr/tarbasev9 (accessed on 18 November 2024), miRTarBase: http://mirtarbase.cuhk.edu.cn/ (accessed on 18 November 2024), and miRBase: https://www.mirbase.org (accessed on 18 November 2024)) for the binding of B7H3 to miRNAs. Upstream miRNAs with the potential to regulate B7H3 protein expression were screened out, including hsa-miR-214-3p, hsa-miR-338-5p, hsa-miR-151a-5p, hsa-mir-17-5p, hsa-mir-130b-5p and hsa-mir-195-5p (Fig. 1D–F).

### The expression of B7H3 and hsa-miR-214-3p negatively correlated in breast cancer, and a dual luciferase reporter gene experiment identified the upstream regulatory miRNA of B7H3 as hsa-miR-214-3p

**Figure 2:**
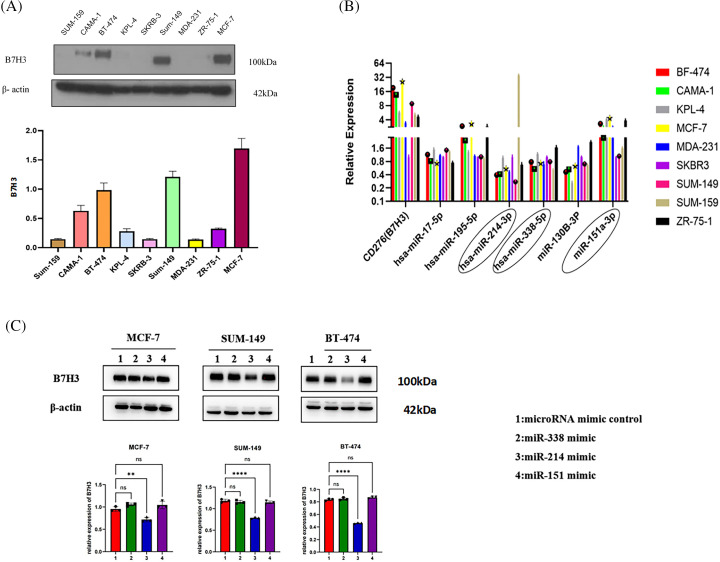
Hsa-miR-214-3p may be the most potential miRNA for upstream regulation of B7H3 expression. (A) The expression of B7H3 in different breast cancer cell lines (it is significantly overexpressed in BT-474, SUM-149, and MCF-7 cells). (B) The expression of hsa-miR-214-3p in different breast cancer cell lines. (C) Western blots of the expression of B7H3 in BT-474, SUM-149, and MCF-7 cells after transfection of hsa-miR-214-3p, hsa-miR-338-5p, and hsa-miR-151-3p mimics. **p < 0.01, ****p < 0.0001. ns, not significant. n = 3 independent experiments.

To further clarify the upstream miRNA of B7H3, we measured the expression of B7H3 and the miRNA to be screened in a variety of breast cancer cells. We found that B7H3 was most highly expressed in the breast cancer cell lines MCF-7, SUM-149, and BT-474, so we carried out follow-up experiments with these cell lines. We observed that the expression of hsa-miR-214-3p, hsa-miR-338-5p, and hsa-miR-151-3p was considerably different from that of B7H3 in different breast cancer cell lines (Fig. 2A,B).

Western blot analysis of B7H3 protein expression demonstrated that after transfection of hsa-miR-214-3p, hsa-miR-338-5p, and hsa-miR-151-3p mimics, the B7H3 protein and mRNA levels in MCF-7, SUM-149, and BT-474 cells changed most significantly with transfection of hsa-miR-214-3p. We speculated that hsa-miR-214-3p may be the most potential miRNA upstream from B7H3 (Fig. 2C).

**Figure 3:**
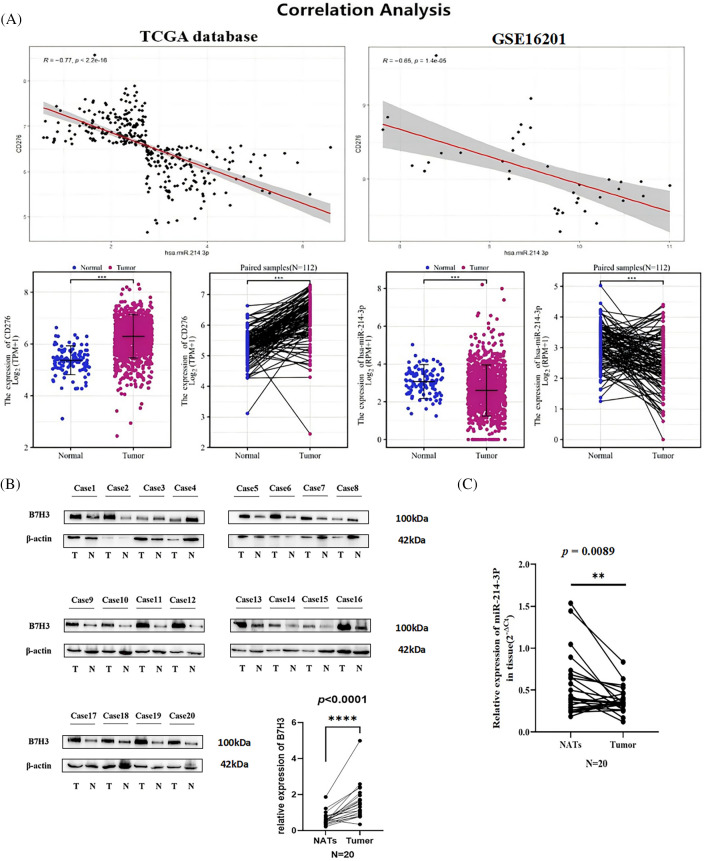
The expression of hsa-miR-214-3p is negatively correlated with the expression of B7H3 in breast cancer cells and samples. (A) Correlation analysis of B7H3 and hsa-miR-214-3p expression in the Cancer Genome Atlas Program (TCGA) and Gene Expression Omnibus (GEO) (GSE16201) data. (B) The expression of B7H3 in cancerous and adjacent normal tissue samples obtained from breast cancer patients (n = 20). (C) The expression of hsa-miR-214-3p in cancerous and adjacent normal tissue samples obtained from breast cancer patients (n = 20). n = 3 independent experiments. **p < 0.01, ***p < 0.001, ****p < 0.0001.

In analyzing the RNA sequences in the TCGA database, we found that the expression of B7H3 in breast cancer samples was higher than that in normal tissue samples. Examination of paired samples of breast cancer samples verified this result (n = 112; *p* < 0.001). In contrast, miRNA sequence analysis of TCGA data demonstrated that the expression of hsa-miR-214-3p in breast cancer samples was lower than that in normal tissue samples, which was also verified in paired samples (n = 112; *p* < 0.001). Subsequently, we further analyzed the correlation between B7H3 and hsa-miR-214-3p expression in TCGA and GEO (GSE16201) data. The results demonstrated that B7H3 expression was negatively correlated with hsa-miR-214-3p expression in both TCGA data (r = −0.77; *p* < 0.001) and GEO data (r = −0.65; *p* < 0.0001) (Fig. 3A). We then collected 20 cancerous and 20 adjacent noncancerous tissue samples from breast cancer patients, extracted RNA and total protein from the samples, detected the expression of B7H3 in them using RT-qPCR and Western blotting, and detected the expression of hsa-miR-214-3p using RT-qPCR alone. We found a negative correlation between the expression of B7H3 and that of hsa-miR-214-3p in the two tissue types: B7H3 expression was increased in cancerous tissue, whereas hsa-miR-214-3p expression was higher in adjacent noncancerous tissue (Fig. 3B,C).

**Figure 4:**
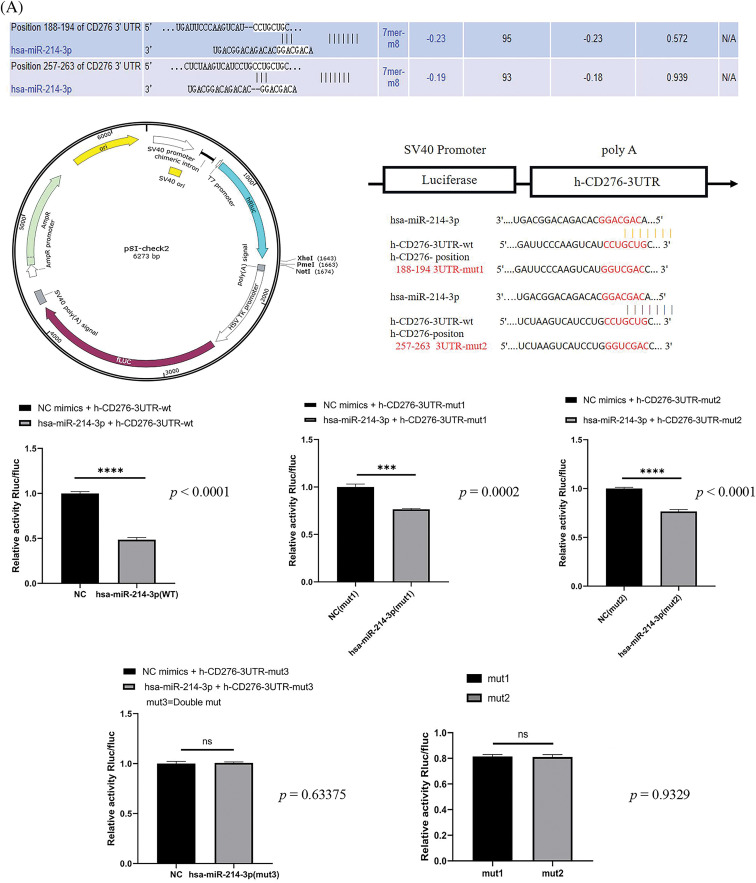

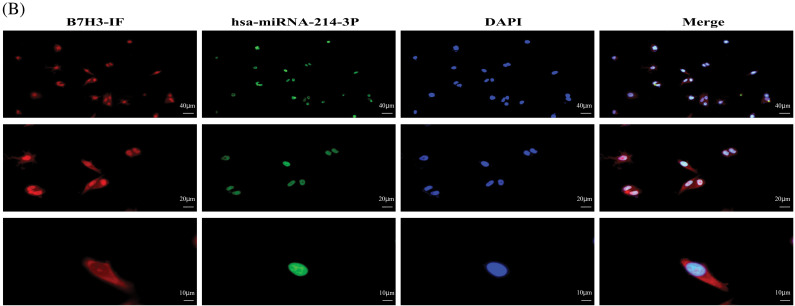
Hsa-miR-214-3p is an upstream regulatory miRNA of B7H3. (A) Dual luciferase reporter gene assay–based detection of the binding sites for B7H3 and hsa-miR-214-3p. ***p < 0.001; ****p < 0.0001. ns, not significant. (B) The subcellular localization of B7H3 and hsa-miR-214-3p in BT-474 as detected using a fluorescence *in situ* hybridization assay. n = 3 independent experiments.

To confirm the regulatory role and sites of hsa-miR-214-3p and B7H3 in breast cancer, we conducted a dual luciferase reporter gene experiment and multiple websites, which identified that hsa-miR-214-3p and B7H3 have two corresponding seven-base sites in 3’UTR of B7H3. Furthermore, we used psiCHECK2 reporter plasmids containing luciferase enzyme and green fluorescent protein (GFP) to construct empty and mutant of B7H3 mRNA constructs for transfection into 293 T cells. The results demonstrated that hsa-miR-214-3p interacted with B7H3 and bound to both seven-base sites, with no difference in binding efficiency between the two sites (Fig. 4A). Given this finding, one can see that hsa-miR-214-3p is the upstream regulatory miRNA for B7H3 and that its binding is stable.

The subcellular localization results for B7H3 and has-miR-214-3p in MCF-7 demonstrated that B7H3 was expressed throughout the entire cell but was mainly concentrated in the cytoplasm and nucleolus, with relatively low expression in the cytoplasm. In comparison, hsa-miR-214-3p was mainly expressed in the nucleus and was almost nonexistent in the cytoplasm. Therefore, B7H3 and hsa-miR-214-3p co-localize in the nucleus (Fig. 4B).

### Negative correlation of B7H3 and hsa-miR-214-3p expression in breast cancer samples and cells

**Figure 5:**
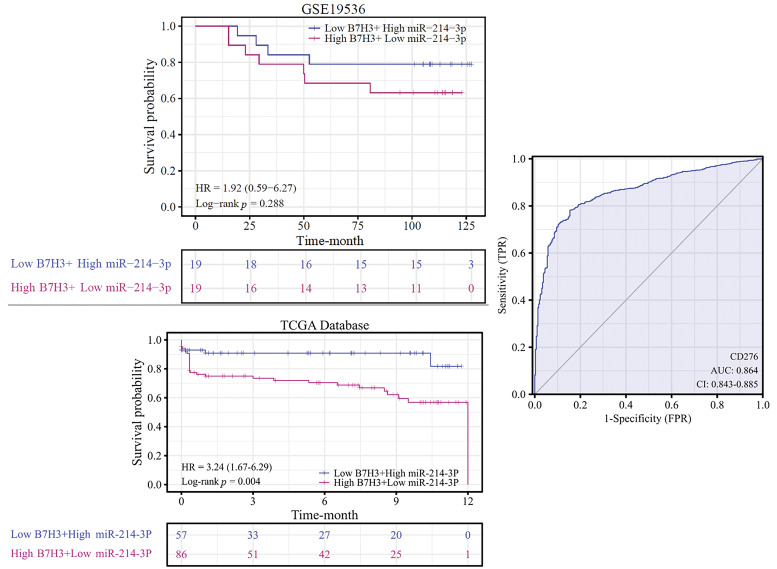
Kaplan-Meier analysis of overall survival in breast cancer patients according to B7H3 and hsa-miR-214-3p expression. The receiver operating characteristic curve shows the value of B7H3 in breast cancer diagnosis for the patients in the Cancer Genome Atlas Program (TCGA) database.

Using Kaplan-Meier analysis, we determined the overall survival rate for breast cancer patients based on the expression of B7H3 and hsa-miR-214-3p in the TCGA and GEO (GSE19536) databases. We placed the patients in two groups based on their B7H3 and hsa-miR-214-expression levels: 1) low expression of B7H3 and high expression of hsa-miR-214-3p and 2) high expression of B7H3 and low expression of hsa-miR-214-3p. The results demonstrated that in the TCGA data, patients with high expression of B7H3 and low expression of hsa-miR-214-3p had worse overall survival than did the other group (hazard ratio, 3.24; *p* = 0.004). In the GEO database, high expression of B7H3 and low expression of hsa-miR-214-3p were also associated with worse overall survival, although the difference was not significant (hazard ratio, 1.92; *p* = 0.288), possibly owing to a small sample size in this database. The receptor operational characteristic curve further confirms that B7H3 can serve as a marker for breast cancer. Specifically, the area under the curve was 0.864, indicating that expression of B7H3 had obviously high sensitivity and specificity in the diagnosis of breast cancer (Fig. 5).

**Figure 6:**
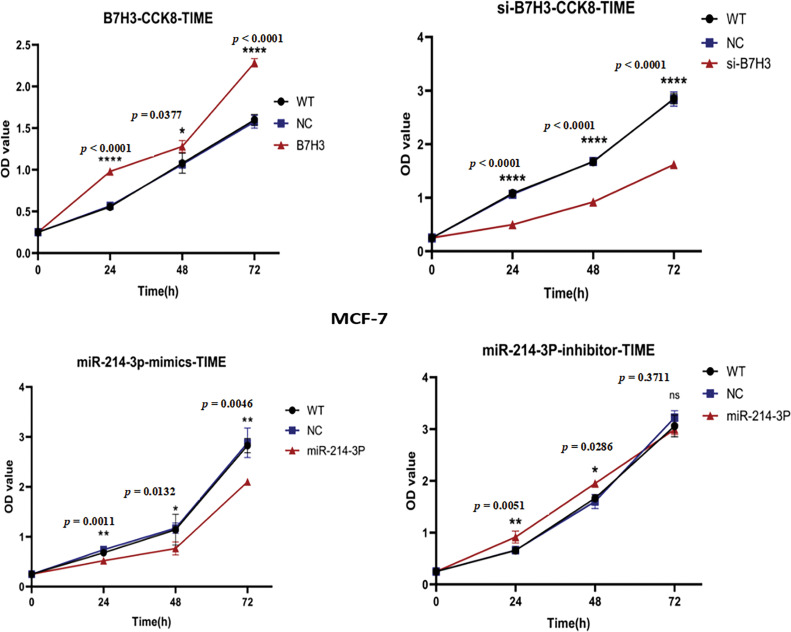
Cell Counting Kit-8 (CCK-8) assay of the proliferation of breast cancer cells. OD, optical density; WT, wild-type. n = 3 independent experiments.

In further *in vitro* experiments, we examined the proliferative ability of MCF-7 cells using the CCK-8 proliferation assay. The results demonstrated that B7H3 overexpression or inhibition of hsa-miR-214-3p expression increased the proliferative ability of these cells, whereas downregulation of B7H3 or transfection of hsa-miR-214-3p mimics significantly inhibited cell proliferation (Fig. 6).

**Figure 7:**
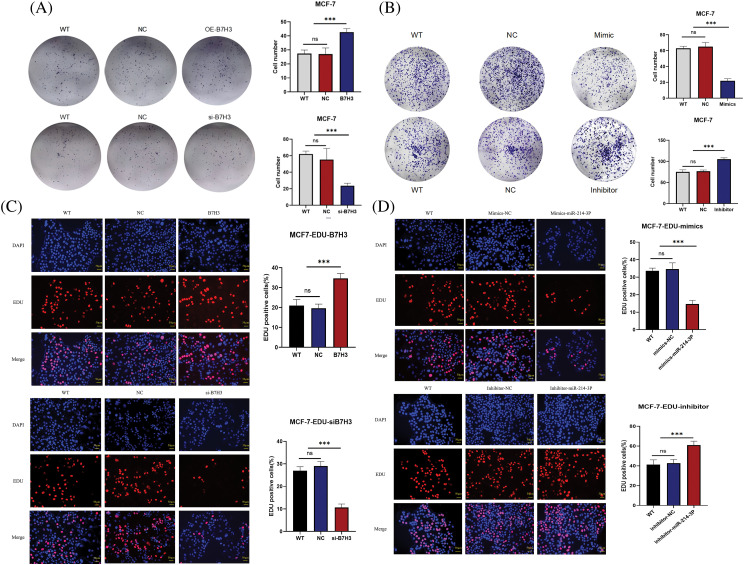
Plate clone and EdU assay. (A and B) Plate clone proliferation assays of breast cancer cells. ***p < 0.001. ns, not significant. (C and D) EdU assay of the proliferation of breast cancer cells. FITC, fluorescein isothiocyanate. ***p < 0.001. ns, not significant. n = 3 independent experiments.

**Figure 8:**
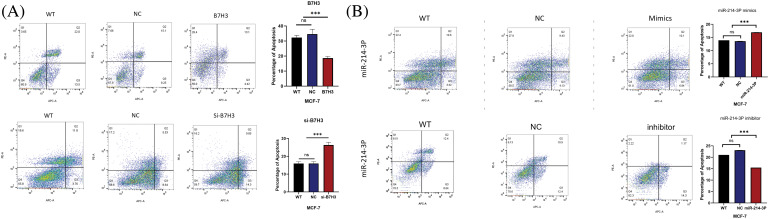
Cell apoptosis assay. (A and B) Breast cancer cell apoptosis capacity assay results. ***p < 0.001. ns, not significant. n = 3 independent experiments.

We validated these results in plate cloning and EdU proliferation experiments (Fig. 7A–D). Also, we used flow cytometry to detect apoptosis of MCF-7 cells and found that B7H3 overexpression or inhibition of hsa-miR-214-3p expression resulted in a decrease in the number of apoptotic MCF-7 cells, whereas cell apoptosis was significantly increased after downregulation of B7H3 or transfection of hsa-miR-214-3p mimics (Fig. 8A,B).

### In vitro experiments confirmed that hsa-miR-214-3p can regulate the expression of B7H3 and its role in promoting breast tumors

**Figure 9:**
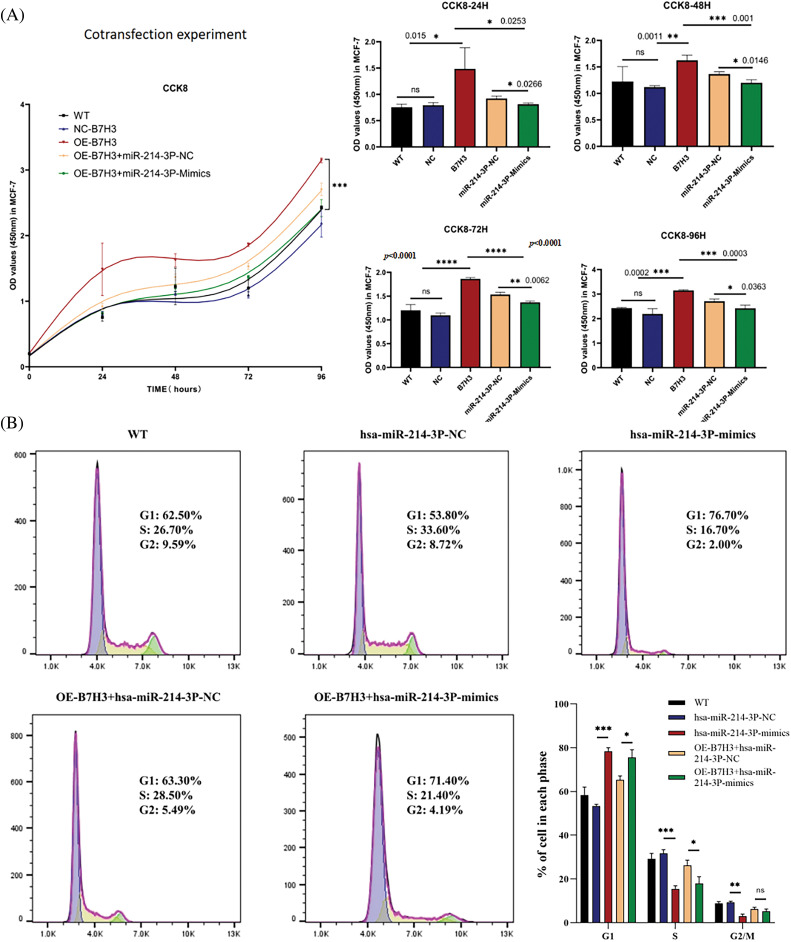
Co-transfection of B7H3 and hsa-miR-214-3p in breast cancer cells demonstrates that hsa-miR-214-3p can regulate breast cancer cell activity by targeting B7H3. (A) Cell Counting Kit-8 (CCK-8) assay of the proliferation of breast cancer cells after co-transfection of B7H3 and hsa-miR-214-3p. OD, optical density; WT, wild-type; ns, not significant. (B) Cell-cycle phases of breast cancer cells transfected with B7H3 plasmid or has-miR-214-3p mimics as detected using flow cytometry. *p < 0.05; **p < 0.01; ***p < 0.001, ****p < 0.0001. ns, not significant. n = 3 independent experiments.

As described above, we used the CCK-8 proliferation assay to determine the proliferative ability of MCF-7 cells. We found that overexpression of B7H3 promoted the proliferative activity of the tumor cells. Based on overexpression of B7H3 in MCF-7, we further transfected hsa-miR-214-3p-mimics and found that cell proliferative ability was inhibited. This indicated that hsa-miR-214-3p can regulate the proliferative activity of MCF-7 by targeting B7H3 (Fig. 9A). In using flow cytometry to assess changes in the cell-cycle progression of MCF-7 cells after hsa-miR-214-3p-mimics transfection, we found that group of overexpression of hsa-miR-214-3p had substantially more cells in G1 phase than did the NC group. Based on overexpression of B7H3 in MCF-7, transfection with hsa-miR-214-3p-mimics resulted in an increase in the number of G1 phase cells and a decrease in the number of S and G2/M phase cells, demonstrating that overexpression of hsa-miR-214-3p can cause cells to stagnate in G1 phase (Fig. 9B).

### Hsa-miR-214-3p improves the immune microenvironment in breast cancer patients by regulating the expression of B7H3

**Figure 10:**
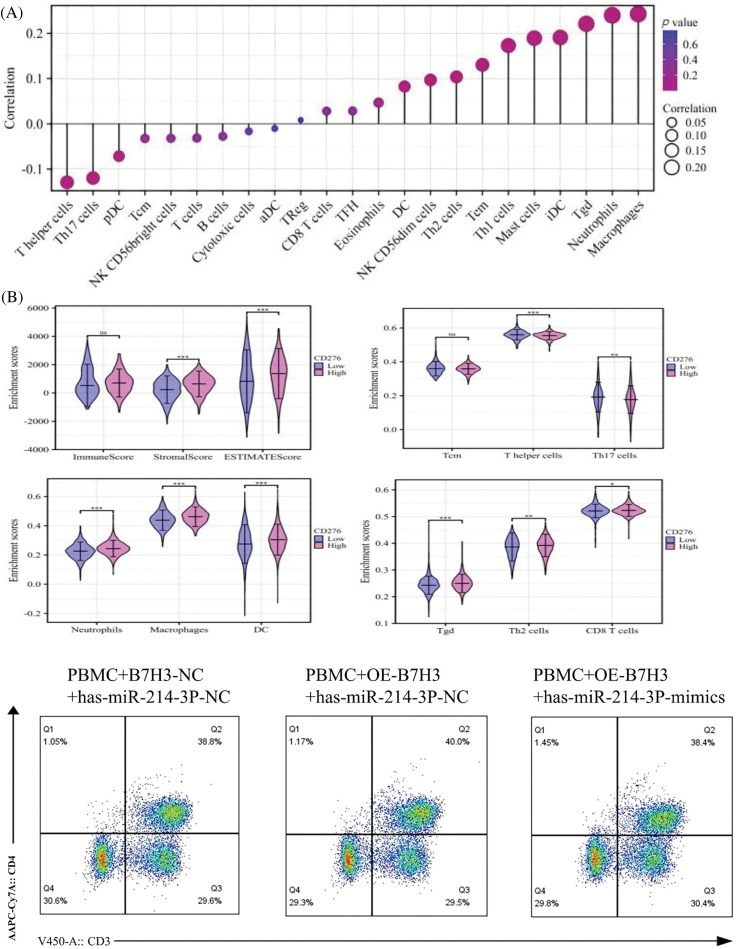

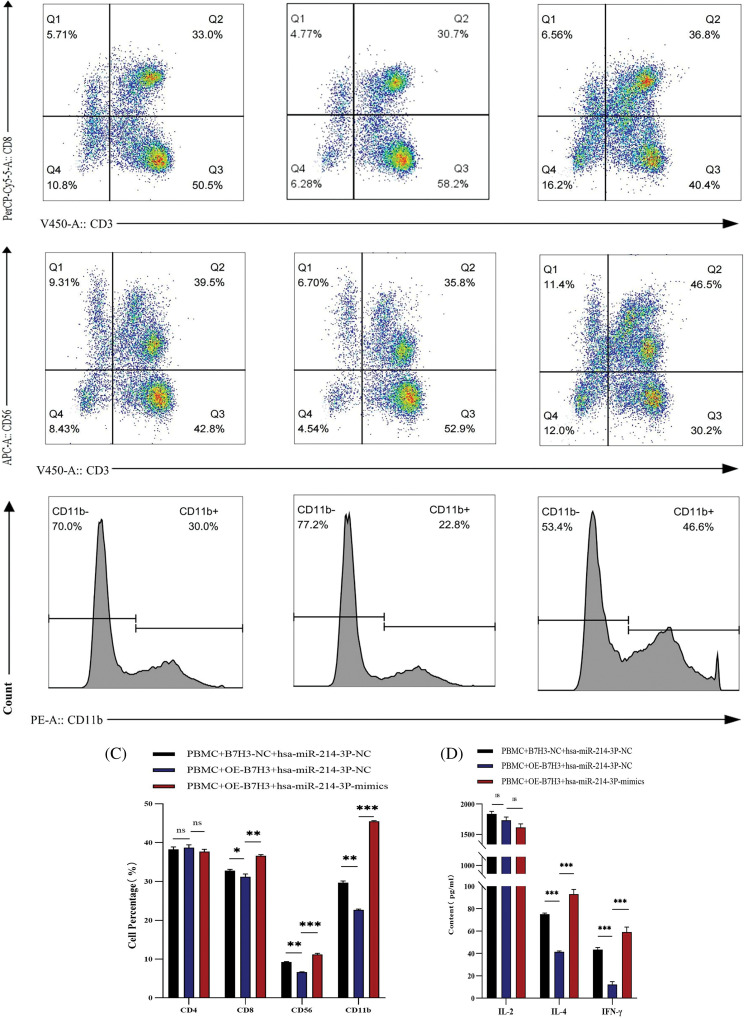

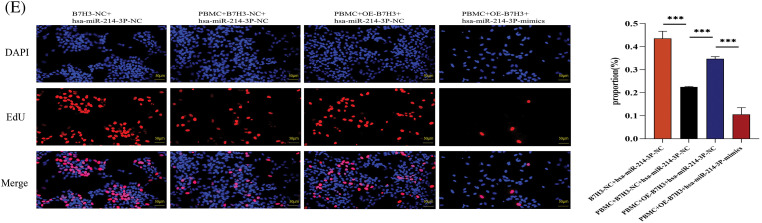
Hsa-miR-214-3p affects the immune microenvironment of breast cancer by regulating B7H3 expression. (A) Correlation of B7H3 expression with infiltration of multiple immune cells in breast cancer samples. (B) Flow cytometric analysis of changes in the proportions of immune cells in peripheral blood mononuclear cells (PBMCs) in the co-culture system of BT-474 and PBMCs. *p < 0.05; **p < 0.01; ***p < 0.001. ns, not significant. (C) Statistical analysis of flow cytometry results above. *p < 0.05; **p < 0.01; ***p < 0.001. ns, not significant. (D) Enzyme-linked immunosorbent assay results showing the levels of IL-2, IL-4, and IFN-γ expression in co-cultured supernatants of BT-474 cells and PBMCs. ***p < 0.001. ns, not significant. (E) EdU assay of the proliferation of in BT-474 in the co-culture system of BT-474 and PBMCs. ***p < 0.001. n = 3 independent experiments in (B, C, D and E).

By analyzing the level of B7H3 immune infiltration in breast cancer samples, we found that B7H3 expression was associated with a variety of immune cells, and these immune cells are closely related to tumor immunity. Specifically, B7H3 expression was positively correlated with macrophages (r = 0.276; *p* < 0.001), neutrophils (r = 0.273; *p* < 0.001), and T cells (r = 0.268; *p* < 0.001) but negatively correlated with T helper cells (r = −0.182; *p* < 0.001) and Th17 cells (r = −0.171; *p* < 0.001). According to the high and low expression groups of B7H3 in the survival analysis of breast cancer patients in the TCGA database, we measured the levels of immune infiltration and extracellular matrix infiltration in these samples. As shown in the lollipop plot, the results demonstrated that high expression of B7H3 indicated high levels of extracellular matrix infiltration. Also, T helper cells and Th17 cells had low levels of extracellular matrix infiltration, whereas the infiltration levels of macrophages, neutrophils, and T cells remained consistent with the results of the lollipop plot (Fig. 10A).

We co-cultured PBMCs extracted from healthy individuals with BT-474 treated with different treatments for 48 h, including empty plasmid and hsa-miR-214-3p-NC, B7H3 overexpression plasmid and hsa-miR-214-3p-NC, B7H3 overexpression plasmid and hsa-miR-214-3p-mimics for 48 h. In using flow cytometry to detect changes of cell number in related immune cells, we found that on the basis of overexpression of B7H3 in BT-474 cells, the group transfected with hsa-miR-214-3p-mimics in together exhibited recovery in the content of CD3^+^CD8^+^ T cells, CD3^-^CD56^+^, and CD11b^+^ cells, whereas the group transfected with hsa-miR-214-3p-NC did not (Fig. 10B,C). At the same time, in ELISA detection of cytokine changes in the supernatants of the co-cultured system, we found that on the basis of overexpression of B7H3 in BT-474 cells, the group transfected with hsa-miR-214-3p-mimics had greater IL-4 and IFN-γ content than did the group transfected with hsa-miR-214-3p-NC (Fig. 10D).

Through detection of the proliferative ability of BT-474 cells in the co-culture with PBMCs using the EdU cell proliferation assay, we found that the proliferation of BT-474 cells not co-cultured with PBMCs was markedly greater than that of co-cultured cells. On the basis of overexpression of B7H3 in BT-474 cells of the co-culture system, the proliferative ability of the group transfected with hsa-miR-214-3p-NC of BT-474 cells was markedly stronger than that of the group transfected with hsa-miR-214-3p-mimics, demonstrating that PBMCs can inhibit the growth of BT-474 cells. Meanwhile, the immunosuppressive function of PBMCs was blocked by overexpression of B7H3, and overexpression of hsa-miR-214-3p inhibited this immunosuppressive effect of B7H3 (Fig. 10E).

## Discussion

The ongoing advancement of immune checkpoint inhibitors as treatments for solid tumors—including melanoma, non-small cell lung cancer, and renal cell carcinoma—has yielded promising therapeutic outcomes and prospects [14]. Breast cancer, however, presents as a highly heterogeneous tumor type with relatively low immunogenicity, and only a select subset of patients exhibit a response to treatments involving immune checkpoint inhibitors such as PD-1 and PD-L1 [15]. In light of this, identifying additional molecular targets beyond PD1 and PD-L1 to enhance the immune response is a critical need in the pursuit of effective immunotherapies for breast cancer. Notably, research has indicated that overexpression of B7H3 contributes significantly to resistance against existing treatments in breast cancer [16]. Research also indicates that B7H3 is overexpressed in various tumors, including breast cancer, non-small cell lung cancer (NSCLC), and ovarian cancer [17]. The overexpression of B7-H3 is closely associated with the invasion, metastasis, proliferation, and prognosis of NSCLC [18] and is linked to the dysfunction of tumor-infiltrating T cells in ovarian cancer [19]. Therefore, our focus is therefore directed towards B7H3, a member of the B7 family which includes PD-L1 (B7-H1), and which has been identified as a potential immune molecular marker in multiple studies [20]. The development of therapeutics targeting B7-H3 represents a viable strategy due to its promising implications [21]. B7H3 may inhibit natural killer (NK) cell activation and exert a proinflammatory effect, triggering cytokine release from monocytes and/or macrophages in a manner dependent on Toll-like receptor-2 and −4 [22]. Additionally, B7H3 is implicated in promoting regulatory T cell activation and in inhibiting NK cell-mediated lysis of glioma cells in patients with non-small cell lung cancer [23]. Beyond its immune-related roles, B7H3 also possesses tumorigenic functions that are non-immune in nature, such as promoting cell migration and invasion, angiogenesis, chemotherapy resistance, and endothelial-to-mesenchymal transition [20]. Furthermore, it impacts tumor cell metabolism. Consequently, the expression of B7-H3 in tumors is associated with a poor prognosis [5].

In this study, utilizing data from the TCGA database, we observed that B7H3 is prominently expressed in breast cancer samples at both transcriptional and protein levels, correlating with poor prognosis—a finding that aligns with those reported by Arigami et al. [24]. Interestingly, while B7H3 mRNA shows widespread expression, B7H3 protein levels remain low in normal tissues [17]. This disparity between cancerous and normal tissues suggests a potential involvement of posttranscriptional mechanisms in regulating B7H3 expression. It has been established that miRNAs play a crucial role in posttranscriptional regulation by forming incomplete complementarity with the 3’ untranslated regions of mRNA, thus influencing gene expression either at the transcriptional or posttranscriptional level. MiRNAs are well-studied biomolecules currently under extensive research [25]. In our analysis, we utilized multiple predictive databases to link this regulatory mechanism to hsa-miR-214-3p. Contrary to previous findings [25], our research uniquely identifies two identical binding sites for hsa-miR-214-3p on B7H3 mRNA. These sites function independently without mutual interference, as evidenced in dual-luciferase reporter assays. Studies have demonstrated that miR-214-3p regulates various cellular processes including proliferation, apoptosis, migration, and invasion [26]. Specifically, overexpression of hsa-miR-214-3p has been shown to inhibit proliferation and invasion in breast cancer cells and to regulate epithelial-mesenchymal transition through the downregulation of RNF8 [27]. In ovarian cancer, increased levels of miR-214-3p correlate with disease progression and reduced disease-free survival [28]. Furthermore, miR-214-3p has been implicated in the regulation of metabolic pathways in breast cancer cells, such as glycolysis and fatty acid synthesis, impacting the energy supply and growth rate of tumor cells [29]. The inhibition of hsa-miR-214-3p has been found to decrease tumor growth and metastasis, positioning it as a promising target for anti-metastatic therapy [30]. Interestingly, no prior studies have explored the interplay between miR-214 and B7H3 in breast cancer. Our findings reveal a negative correlation between the expression and function of hsa-miR-214-3p and B7H3 in this malignancy. Specifically, hsa-miR-214-3p is expressed at lower levels in tumors and at higher levels in normal tissues, whereas B7H3 exhibits the opposite pattern. Overexpression of hsa-miR-214-3p inhibits, while overexpression of B7H3 promotes, tumor proliferation. Further experimental data confirm that overexpressing hsa-miR-214-3p diminishes B7H3 expression, thereby reducing the proliferative capacity of tumor cells.

Previous research has shown that hsa-miR-214-3p regulates the function and characteristics of various immune cells, including dendritic cells, T cells, NK cells, and macrophages, playing a significant role in the immune response [31]. Additionally, the modulation of B7H3 by hsa-miR-214-3p has been associated with tumor immune escape. Li et al. [32] reported that LINC01123 could facilitate immune escape by sequestering miR-214-3p to regulate B7H3 in head and neck squamous cell carcinoma. In our current study, we co-cultured peripheral blood mononuclear cells (PBMCs) from healthy individuals with BT-474 cells, which exhibit high B7H3 expression and overexpression of hsa-miR-214-3p. Our findings indicated that the populations of CD8, CD56, and CD11b-positive cells in the PBMCs were significantly larger than those in PBMCs co-cultured with BT-474 cells that only overexpressed B7H3. However, the populations of CD4-positive cells showed no significant differences. Furthermore, the levels of secreted immune cytokines such as IL-2, IL-4, and IFN-γ were markedly increased in the supernatant of the co-culture system. This suggests that hsa-miR-214-3p can counteract the suppressive effect of B7H3 on CD8^+^ T cells and NK cell-dominated immune responses in BT-474 cells. Our study confirms that hsa-miR-214-3p regulates B7H3 expression and can mitigate the inhibitory impact of B7H3 on immune cells. This implies that in breast cancer cells, enhancing the expression of hsa-miR-214-3p improves the activity and proportion of immune cells through the regulation of B7H3, thus obstructing the immune escape of breast cancer cells.

In conclusion, our research establishes that hsa-miR-214-3p can modulate the expression of B7H3 in breast cancer cells, thereby inhibiting tumor cell proliferation, reinvigorating immune cell activity, and ameliorating the immune microenvironment. We have identified two distinct sites where hsa-miR-214-3p exerts similar regulatory effects on B7H3, providing novel insights into the role of hsa-miR-214-3p in B7H3 regulation and laying a robust theoretical foundation for the clinical application of targeting either B7H3 or hsa-miR-214-3p in breast cancer therapies. However, this study has certain limitations: the receptors and associated binding partners of B7H3 have not been precisely identified [5], which constrains the development of targeted therapies for B7H3. Hence, further investigation into the receptor and its binding partners is warranted. Proliferation and apoptosis experiments related to B7-H3 have shown promising results in laboratory settings, but these methods have certain limitations. For example, these experiments are typically conducted under idealized *in vitro* conditions, which may not fully replicate the complex *in vivo* environment. Additionally, the cell lines used in the experiments may not accurately represent the diverse patient population or real pathological conditions. Moreover, the regulatory pathway involving the hsa-miR-214-3p/B7H3/immune cell axis requires further elucidation. Future research will utilize co-culture systems, *in vivo* experiments, and single-cell RNA sequencing to explore the specific mechanisms by which hsa-miR-214-3p and B7H3 influence breast cancer and immune cells.

## Conclusions

This study substantiated the hypothesis that B7H3 plays a pivotal role in the immunosuppression observed in breast cancer and demonstrated that hsa-miR-214-3p can effectively modulate its expression. Our principal findings indicate that targeting B7H3 with hsa-miR-214-3p not only inhibits the proliferation of breast cancer cells but also augments the immune response, thereby presenting a viable therapeutic strategy. These results enrich our understanding of the mechanisms behind immune evasion in breast cancer and underscore potential therapeutic targets. Nevertheless, the specific immune cells affected by the hsa-miR-214-3p/B7H3 axis in the context of breast cancer remain to be precisely identified. Future research should prioritize detailed mechanistic studies and the initiation of clinical trials to corroborate these findings and to further the development of personalized therapeutic strategies.

## Data Availability

All data generated or analyzed during this study are included in this published article.
